# Health-related quality of life in stroke survivors: a 5-year follow-up of The Fall Study of Gothenburg (FallsGOT)

**DOI:** 10.1186/s12877-023-04308-z

**Published:** 2023-09-22

**Authors:** Mårten Segerdahl, Per-Olof Hansson, Carina M. Samuelsson, Carina U. Persson

**Affiliations:** 1 Department of Internal Medicine, Nyköping Hospital, Region Sörmland, Nyköping, Sweden; 2 Department of Molecular and Clinical Medicine, Institute of Medicine, Sahlgrenska Academy, University of Gothenburg, Gothenburg, Sweden; 3 Department of Medicine, Geriatrics and Emergency Medicine, Sahlgrenska University Hospital, Gothenburg, Region Västra Götaland Sweden; 4 Department of Occupational Therapy and Physiotherapy, Sahlgrenska University Hospital/Östra, Gothenburg, Region Västra Götaland Sweden; 5 Department of Clinical Neuroscience, Rehabilitation Medicine, Institute of Neuroscience and Physiology, Sahlgrenska Academy, University of Gothenburg, Vita Stråket 12, S-413-45 Gothenburg, Sweden

**Keywords:** Stroke, Quality of life, Health status, Rehabilitation

## Abstract

**Background:**

There are limited data on long-term prevalence and predictors of health-related quality of life (HRQoL) in stroke survivors. Therefore, the aim was to describe the prevalence of impaired HRQoL, and to identify factors in acute stroke that are associated with impaired HRQoL, 5 years after stroke.

**Methods:**

The 305 (60.5%) stroke survivors of the original 504 participants from The Fall Study of Gothenburg were invited to participate in a 5-year follow-up including assessment of HRQoL using the EuroQol 5 Dimensions 3 Levels questionnaire (EQ-5D-3L). To identify baseline predictors of impaired HRQoL, based on the EQ-5D-3L’s five dimensions, univariate and multivariate logistic regression analyses were performed.

**Results:**

A total of 129 participants (42.3% of the survivors) completed the questionnaire at a median follow-up time of 58 months. At baseline, their mean age was 70.6 years, and they had a median NIHSS score of 1. The median (interquartile range [IQR]) EQ-5D-3L index score was 0.87 (0.71–0.93) and the median (IQR) EQ-visual analogue scale was 70 (49.8–88). In total, 102 (79%) participants were classified as having impaired HRQoL, stated primarily (among 68.5%) related to Pain/Discomfort. Higher age was identified as a predictor of impaired HRQoL related to Mobility (Odds ratio (OR) 1.05, 95% confidence interval (CI) 1.01–1.10) and Self-Care (OR 1.09, 95% CI 1.02–1.17), and longer hospital stay at baseline was identified as a predictor related to Mobility (OR 1.09, 95% CI 1.01–1.18), Self-Care (OR 1.10, 95% CI 1.02–1.18)) and Usual Activities. (OR 1.10, 95% CI 1.03–1.18).

**Conclusion:**

At 5 years after stroke, four out of five participants (79%) reported impaired HRQoL related to any of the five dimensions assessed by using the EQ-5D-3L. Most reported, impaired HRQoL was related to Pain/Discomfort. Higher age and longer hospital care period at index stroke were associated with impaired HRQoL. The findings could assist to identify individuals at high risk of low HRQoL, who might benefit from special attention and psychological support.

## Introduction

Following a stroke, chronic negative effects on physical, mental and social functions are common [1]. A key outcome in stroke care is health-related quality of life (HRQoL) [2]. HRQoL has been defined as "an individual’s or group’s perceived physical and mental health over time” [3], and focuses on the importance of health status on quality of life (QoL) [2]. Understanding which factors are associated with HRQoL in stroke survivors may assist clinicians and policymakers in actions aiming to improve HRQoL during rehabilitation and life after stroke, but long-term follow-ups of HRQoL are limited.

To address the research gaps with limited data on long-term prevalence and predictors of HRQoL in stroke survivors, the aim of this study was to describe the prevalence of impaired HRQoL, and to identify factors in acute stroke that are associated with impaired HRQoL, 5 years after stroke. Based on previous research on factors associated with impaired HRQoL regardless of timepoint after stroke and clinical reasoning, we hypothesised that impaired HRQoL is common among stroke survivors 5 years after stroke and that older age [4, 5], female sex [5, 6], higher stroke severity [7], longer duration of hospital stay [4] and cardiovascular comorbidities would all be associated with impaired self-reported HRQoL.

## Materials & methods

### Study design

The current study formed a follow-up of the participants in The Fall Study of Gothenburg (FallsGOT) [8], a cross-sectional and longitudinal observational cohort study, 5 years following enrolment at index stroke. Comprehensive study details of the FallsGOT’ eligibility criteria, the methods and participant characteristics have been described in detail elsewhere [8–10]. In brief, the study comprised consecutively included patients from one of three stroke units at the Sahlgrenska University Hospital in Gothenburg, Sweden who had a clinical diagnosis of stroke including infarction, intracerebral hemorrhage, and subarachnoid hemorrhage. People who were unwilling to participate in the study or those with a palliative condition for whom the patient’s physician deemed inclusion unethical were excluded from consideration. None of the included patients got intervention with thrombolysis or thrombectomy, those patients considered to need that treatment were referred to another stroke unit in the city. At the inclusion at baseline, the median age was 77 years old and the median NIHSS score was 2. The baseline variables were assessed during the first four days after admission to the stroke unit. Stroke was defined as a clinical diagnosis of stroke, i.e., it had been registered with any of the International Classification of Diseases (ICD-10) codes I60, I61 and I63. Participants who completed the EuroQol 5 Dimensions 3 Levels questionnaire (EQ-5D-3L) [11] at follow-up were included in the present study.

The FallsGOT has been registered at ClincialTrials.gov (identifier: NCT02264470; 15/10/2014). The Regional Ethical Review Board of Gothenburg approved the original study (reference number 004–14) and the Swedish Ethical Review Authority approved the 5-year follow-up (reference numbers: 2019–06476). Prior to the start of the original study, written informed consent was obtained from all the participants, or from the patients’ next of kin if a patient was unable to read or understand the information.

### Assessment at baseline

The variables assessed at index stroke were available from the FallsGOT database and included: age; sex; length of stay (LOS); fear of falling; the National Institutes of Health Stroke Scale (NIHSS) [12]; body mass index (BMI); self-reported previous physical activity level (using the Saltin-Grimby Physical Activity Level Scale [SGPALS]) [13, 14]; history of stroke; presence of comorbidity (hypertension; diabetes mellitus; atrial fibrillation; congestive heart disease or ischemic heart disease), number of medications and use of antidepressant medication (Anatomical Therapeutic Chemical [ATC] code N06A). The Oxford Classification of Stroke [15] was used to classify ischemic strokes according to subtype. In the current study, the independent variables (i.e., the potential predictors) were defined as age, female sex, LOS, fear of falling, NIHSS, diabetes mellitus and ischemic heart disease.

### Assessment of health-related quality of life at 5 years after baseline

Those who were still alive in August 2020 (4 to 6 years following their inclusion in the FallsGOT) were mailed an invitation to participate in the current study, excluding individuals with unknown address and withdrawn consent. Non-responders received a postal reminder after one month. The participants were asked to provide their current health state, i.e., HRQoL (the dependent variable), in the EQ-5D-3L for five different dimensions of health; Mobility, Self-Care, Usual Activities (e.g., work, household chores, family and leisure activities), Pain/Discomfort and Anxiety/Depression by using one of three response categories. Level 1 (1) indicates no problems, level 2 (2) indicates moderate problems and level 3 (3) indicates severe problems. Accordingly, based on the five dimensions with the three response categories each, a total of 243 unique health profiles can potentially be described. A health profile of 11111 indicates no problems on any of the dimensions, while a health profile of 33333 indicates severe problems on all the dimensions. EQ-5D-3L also includes a visual analogue scale (EQ-VAS), in which the participant approximates her/his current state of health on a graded scale anchored at 0 (the worst imaginable state of health) and 100 (the best imaginable state of health). The EQ-5D-3L has previously been found to be a valid and reliable tool for the assessment of HRQoL in individuals suffering mild to moderate functional limitations due to stroke and to have a reasonable responsiveness in functional status following stroke [16].

### Statistical analyses

Descriptive data on participant characteristics and data derived from the EQ-5D-3L questionnaire and the EQ-VAS were presented as means with standard deviations (SDs) and medians and inter-quartile ranges (IQRs, more specifically Q1 and Q3) as appropriate and summarized using number and percent per each response category. Each health profile was converted into a corresponding EQ-5D-3L value index by using the Swedish Time Trade Off data set based on pooled data from 49,169 individuals collected in 2004 and 2006 from the general population in Skåne and Stockholm counties, Sweden [17].

Univariate logistic regression analyses were used to assess the association between the five dependent variables, i.e., the five dimensions in the EQ-5D-3L: Mobility, Self-Care, Usual Activities, Pain/Discomfort and Anxiety/Depression, and the independent variables: age, female sex, LOS, fear of falling, NIHSS, diabetes mellitus and ischemic heart disease at index stroke. The EQ-5D-3L results were dichotomized, as is considered conventional [18], with the proportion of participants who had any problem (moderate or severe, i.e., level 2 or 3) or no problem reported (i.e., level 1). Spearman´s rank correlation analyses were used to examine any presence of multicollinearity between the statistically significant independent variables. Correlation coefficients of ≥ 0.7 were considered as multicollinear [19]. Variables that were statistically significantly (*p*-value < 0.1) associated with reporting problems in an EQ-5D-3L dimension in the univariate analysis were entered into a multivariate logistic regression analysis for this specific EQ-5D-3L dimension. Goodness-of-fit was tested using the Homer-Lemeshow test, where a non-significant value indicates a good fit of the data against the model. All models were then evaluated using the Cox & Snell and Nagelkerke Pseudo-*R*^*2*^ values. The models’ discriminatory ability was assessed by plotting a receiver operating characteristic (ROC) curve, and an area under the curve (AUC) was reported. The AUC takes a value between 0 and 1, where 0 indicates zero accuracy and 1 indicates perfect accuracy. An AUC of 0.7 to 0.8 was considered acceptable, 0.8 to 0.9 was considered excellent and more than 0.9 was considered outstanding [20].

Results were presented as odds ratios (ORs), 95% confidence intervals (CIs) and *p*-values. The statistical significance level in the final multivariate analyses was set as a two-sided *p*-value at < 0.05. All statistical analyses were performed using the Statistical Package for the Social Sciences (SPSS, IBM Corp. Released 2021. SPSS Statistics for Windows, Version 27.0. Armonk, NY: IBM Corp).

## Results

**Fig. 1:**
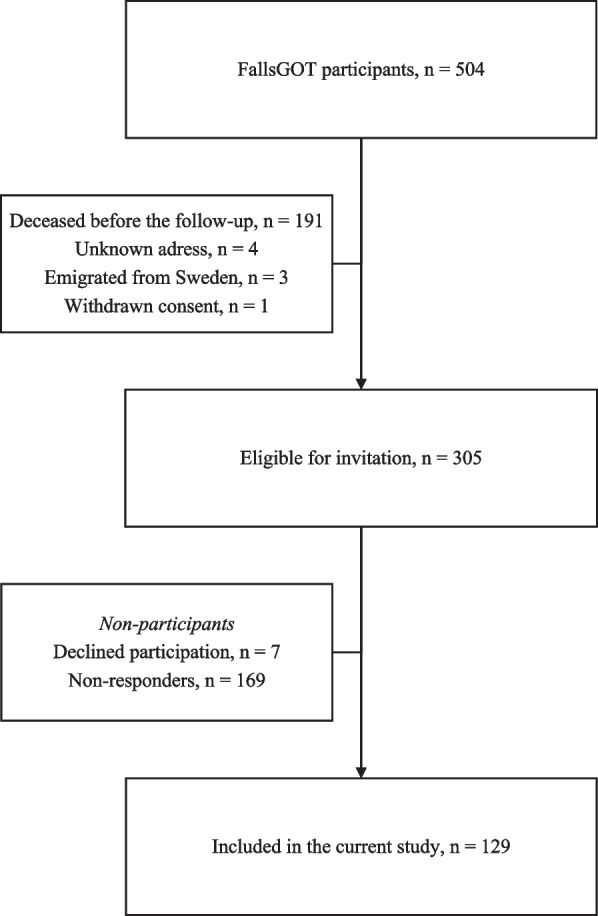
Flow-chart

Of the 305 stroke survivors invited to the follow-up, the EQ-5D-3L questionnaire was responded by 129 (42.3%) (123 with cerebral infarction, six with intracerebral hemorrhage, and none with subarachnoid hemorrhage). Among these participants, 127 completed the EQ-5D-3L and 109 completed the EQ-VAS scale. Figure 1 outlines the flow-chart from the inclusion.

### Sample characteristics

The median (IQR) and min–max time for the follow-up was 58 (52–63) and 49–70 months after index stroke. Clinical characteristics of the participants and non-participants at baseline and EQ-5D-3L index value set (score) and EQ-VAS at 5-year follow-up are presented in Table 1. Most study participants were men (55.8%) and one in three were not physically active prior the stroke. The most common subtype was a posterior circulation stroke. At baseline, non-participants were slightly older, had more severe stroke, they were more commonly on antidepressant medication and one out of every four were current smokers.

**Table 1:** Clinical characteristics at baseline for the participants and the non-participants, and EQ-5D-3L index and EQ-VAS at the 5-year follow-up, for the participants, per quartiles of EQ-VAS

Clinical characteristic	**Total, *n* = 129**	**EQ-VAS < = 48, *n* = 28**	**EQ-VAS 49–69, *n* = 28**	**EQ-VAS 70–80, *n* = 29**	**EQ-VAS 81 + , *n* = 24**	**Missing EQ-VAS, *n* = 20**	**Non-participants, *n* = 176**
**Baseline**							
Age, years, mean (SD)	70.6 (10.4)	75.1 (10.5)	72.5 (9.4)	69.5 (9.6)	65.3 (9.8)	69.9 (10.6)	73.1 (12.0)
Women, n (%)	57 (44.2)	11 (39.3)	13 (46.4)	11 (37.9)	14 (58.3)	8 (40.0)	85 (48.3)
BMI, median kg/m^2^ (IQR)	25.8 (23.5–29.1)	28.2 (24.4–31.7)	25.0 (23.0–27.9)	26.3 (24.2–28.7)	24.3 (22.6–27.1)	27.0 (22.5–30.3)	26.3 (23.5–29-3)
LOS, days, median (IQR)	6 (4–9)	8 (4.3–12.5)	5.5 (4.3–9.0)	5 (3–7.5)	6.5 (4–11.3)	6 (3–13.8)	7.5 (4–14.8)
Fear of falling, n (%)	40 (31.7), *n* = 126	8 (28.6)	8 (28.6)	8 (27.6)	7 (30.4), *n* = 23	9 (45.0)	87 (52.4), *n* = 166
NIHSS, median score (IQR)	1 (0–2), *n* = 125	2 (0.3–4)	1 (0–2)	0 (0–2)	0 (0–1), *n* = 22	1 (0–2), *n* = 19	2 (0.75–4), *n* = 174
Cerebral infarction, n (%)	123 (95.3)	25 (89.3)	27 (96.4)	29 (100)	24 (100)	18 (90.0)	162 (92.0)
Oxford Classification, n (%)							
TAC	3 (2.4)	2 (8.0)	1 (3.7)	0	0	0	3 (1.7), *n* = 175
PAC	39 (31.7)	10 (40.0)	8 (29.6)	7 (24.1)	5 (20.8)	9 (50.0)	56 (32.0), *n* = 175
LAC	37 (30.0)	6 (24.0)	6 (22.2)	14 (48.3)	7 (29.2)	4 (22.2)	66 (37.7), *n* = 175
POC	44 (35.8)	7 (28.0)	12 (44.4)	8 (27.6)	12 (50)	5 (27.8)	50 (28.6), *n* = 175
SGPALS, n (%)							
Physically inactive	43 (33.3)	15 (53.6)	6 (21.4)	9 (31.0)	5 (20.8)	8 (40.0)	103 (59.2), *n* = 174
Some light physical activity	75 (58.1)	13 (46.4)	22 (78.6)	16 (55.2)	13 (54.2)	11 (55.0)	64 (36.8), *n* = 174
Regular physical activity	11 (8.5)	0	0	4 (13.8)	6 (25.0)	1 (5.0)	7 (4.0), *n* = 174
Hard physical training	0	0	0	0	0	0	0
Ischemic heart disease, n (%)	24 (18.6)	8 (28.6)	5 (17.9)	4 (13.8)	2 (8.3)	5 (25.0)	39 (22.2)
Current smoker, n (%)	21 (17.2), *n* = 122	6 (21.4)	4 (16.0), *n* = 25	5 (18.5), *n* = 27	2 (9.1), *n* = 22	4 (20.0)	43 (25.9), *n* = 166
Use of antidepressants, n (%)	11 (8.5)	5 (17.9)	3 (10.7)	1 (3.4)	1 (4.2)	1 (5.0)	34 (19.3)
Congestive heart failure, n (%)	9 (7.0)	3 (10.7)	3 (10.7)	1 (3.4)	0	2 (10.0)	13 (7.4)
Number of drugs, mean (SD)	5.5 (2.7)	6.4 (2.9)	5.7 (2.2)	5.2 (3.2)	4.3 (2.2)	5.9 (2.3)	6.7 (3.5)
**Five-year follow-up**							
EQ-5D-3L index, median (IQR)	0.87 (0.71–0.93)	0.64 (0.58–0.77)	0.81 (0.71–0.93)	0.87 (0.81–0.97)	0.97 (0.90–0.97)	0.88 (0.73–0.93)	-
EQ-VAS, median (IQR)	70 (49.8–88)	39.5 (25–40.8)	60.0 (51.3–65)	75.0 (71–80)	90.0 (89–92.3)	-	-

*Abbreviations*: *EQ-5D-3L* EuroQol 5 Dimensions 3 Levels, *EQ-VAS* EuroQol Visual Analogue Scale, *BMI* Body Mass Index, *LOS* Length of stay, *NIHSS* The National Institute of Health Stroke Scale, *TAC* Total anterior circulation stroke, *PAC* Partial anterior circulation stroke, *LAC* Lacunar stroke, *POC* Posterior circulation stroke, *SGPALS* Saltin-Grimby Physical Activity Level Scale

The mean (SD) EQ-5D-3L index was 0.82 (± 0.14). The median (IQR) EQ-5D-3L index was 0.87 (0.71–0.93) with a range between 0.43 and 0.97. For women and men, the median EQ-5D-3L index score was 0.87 (IQR 0.71–0.94) and 0.85 (IQR 0.76–0.93), respectively.

The median EQ-VAS for women and men, respectively, was 75 (IQR 50–89) and 70 (IQR 47.5–80). The mean (SD) EQ-VAS reported for all the participants was 63.8 (± 22.4). A total of 27 participants (20.9%), 13 women and 14 men, reported a health profile of 11111, i.e., “perfect” health status. None of the participants reported a health profile of 33333, i.e., “worst possible” health status.

Age at baseline ranged between 41 and 91 years. The mean (SD) age of the participants at the follow-up, when completed the EQ-5D-3L questionnaire, was 75.6 (± 10.4) years (not shown in table).

### Outcome data

A summary of the EQ-5D-3L data in terms of numbers and frequencies of reported problems by level and dimension for the 129 participants is presented in Table 2. The most frequently reported issues were related to the dimension Pain/Discomfort, affecting two-thirds of the participants, and the dimensions Mobility and Anxiety/Depression, reported by more than half of the population, respectively. Having severe problems was especially reported (by every tenth participant) related to the dimension Usual-Activities, closely followed by the dimension Pain/Discomfort.

**Table 2:** Summary of the EQ-5D-3L data as numbers and frequencies of reported problems by each level and dimension, for the 129 participants

EQ-5D-3L Response category	Mobility	Self-Care	Usual Activites	Pain/Discomfort	Anxiety/Depression
	***N* = 127**	***N* = 127**	***N* = 129**	***N* = 127**	***N* = 129**
	***N* (%)**	***N* (%)**	***N* (%)**	***N* (%)**	***N* (%)**
Level 1, no problem	60 (47.2)	110 (86.6)	87 (67.5)	40 (31.5)	64 (49.6)
Level 2, moderate problems	61 (48.0)	10 (7.9)	29 (22.5)	75 (59.1)	58 (45.0)
Level 3, severe problems	6 (4.7)	7 (5.5)	13 (10.1)	12 (9.4)	7 (5.4)
Level 2 or 3, moderate or severe problems	67 (52.8)	17 (13.4)	42 (32.6)	87 (68.5)	65 (50.4)

*Abbreviations*: *EQ-5D-3L* EuroQol 5 Dimensions 3 Levels

In the univariate logistic regression analysis, shown in Table 3, four of the independent variables (age, female sex, LOS and ischemic heart disease) were associated with reporting problems in one or more dimensions of the EQ-5D-3L. Since only one of the potential predictors associated with the dimension Pain/Discomfort and none of the potential predictors associated with the dimension Anxiety/Depression, multivariate logistic regression analyses, shown in Table 4, were only performed for the other three of the five EQ-5D-3L dimensions. Higher age and longer LOS were statistically significant predictors of impaired HRQoL related to the dimension Mobility in the multivariate model. Higher age and longer LOS were also associated with impaired HRQoL related to Self-Care, while only LOS was found to be a significant predictor of impaired HRQoL related to the multivariate model for Usual-Activities. The multivariate model for Self-Care showed acceptable discriminatory power.

**Table 3:** Univariate logistic regression analysis with odds ratios (ORs) and 95% confidence intervals (CIs) and *p*-values for prediction of impaired health-related quality of life assessed by using each of the five dimensions in the EQ-5D-3L

	**Mobility**		**Self-Care**		**Usual Activities**		**Pain/Discomfort**		**Anxiety/Depression**	
**Independent variables**	**OR (95% CI)**	**p**	**OR (95% CI)**	**p**	**OR (95% CI)**	**p**	**OR (95% CI)**	**p**	**OR (95% CI)**	**p**
Higher age	1.054* (1.015–1.094)	0.006	1.093* (1.027–1.164)	0.005	1.039* (1.000–1.080)	0.048	1.032* (0.995–1.070)	0.094	0.996 (0.963–1.030)	0.807
Female	0.872 (0.433–1.757)	0.702	0.356* (0.109–1.160)	0.087	1.229 (0.586–2.574)	0.586	0.673 (0.317–1.430)	0.303	1.036 (0.517–2.075)	0.921
Longer LOS	1.091* (1.015–1.173)	0.019	1.102* (1.028–1.181)	0.006	1.101* (1.029–1.178)	0.005	1.007 (0.947–1.069)	0.832	1.044 (0.983–1.109)	0.158
Fear of falling	0.594 (0.273–1.291)	0.189	0.475 (0.159–1.420)	0.183	0.950 (0.426–2.112)	0.901	0.741 (0.316–1.736)	0.490	0.673 (0.316–1.435)	0.306
NIHSS	1.002 (0.982–1.023)	0.826	0.997 (0.964–1.030)	0.840	0.985 (0.954–1.017)	0.354	0.992 (0.972–1.012)	0.435	0.989 (0.967–1.012)	0.337
Diabetes mellitus	0.800 (0.324–1.978)	0.629	2.298 (0.712–7.418)	0.164	0.552 (0.219–1.393)	0.208	1.881 (0.644–5.493)	0.248	0.712 (0.287–1.769)	0.465
Ischemic heart disease	1.875 (0.732–4.801)	0.190	2.788* (0.914–8.507)	0.072	1.214 (0.461–3,201)	0.695	1.826 (0.626–5.329)	0.270	1.019 (0.420–2.474)	0.966

*Abbreviations*: *EQ-5D-3L* EuroQol 5 Dimensions 3 Levels, *LOS* Length of stay, *NIHSS* the National Institute of Health Stroke Scale

^*^significant at *p*-value < 0.1

**Table 4:** Multivariate logistic regression with odds ratios (ORs) and 95% confidence intervals (CIs) for prediction of impaired health-related quality of life assessed by using three of the five dimensions in the EQ-5D-3L

	**Mobility** ***N* = 127**		**Self-Care** ***N* = 127**		**Usual Activities** ***N* = 129**	
**Independent variables**	**OR (95% CI)**	**p**	**OR (95% CI)**	**p**	**OR (95% CI)**	**p**
Higher age	1.053* (1.014–1.095)	0.008	1.093* (1.023–1.168)	0.008	1.039 (0.998–1.081)	0.063
Longer LOS	1.092* (1.011–1.179)	0.025	1.098* (1.021–1.180)	0.011	1.101* (1.027–1.181)	0.007

Area under the curve (with 95% confidence interval and *p*-value) for each model

Mobility 0.689 (0.596–0.782, *p* < 0.001), Self-Care 0.763 (0.635–0.892, *p* < 0.001) and Usual Activities 0.654 (0.547–0.761, *p* = 0.005)

Hosmer and Lemeshow test, *p*-value for each model

Mobility 0.361, Self-Care 0.862, Usual Activities 0.806

Cox & Snell and Nagelkerke R square for each model

Mobility 0.109 and 0.145, Self-Care 0.121 and 0.222, Usual Activities 0.097 and 0.136

*Abbreviations*: *EQ-5D-3L*: EuroQol 5 Dimensions 3 Levels, *LOS* Length of stay

^*^significant at *p*-value < 0.05

## Discussion

In this observational 5-year follow-up study, four out of five participants were classified as having impaired HRQoL. The median EQ-5D-3L index score was 0.87 and the median EQ-VAS was 70. According to the EQ-5D-3L dimension Mobility, more than half of the stroke survivors were classified as having impaired HRQoL, and more than two thirds reported impaired HRQoL according to the EQ-5D-3L dimension Pain/Discomfort. For the dimensions Mobility and Self-Care, a lower HRQoL was associated with a higher age, and for the dimensions Mobility, Self-Care and Usual Activities, with a longer care time. Our hypothesis that higher age and longer LOS were predictors of HRQoL was confirmed. There was no association between HRQoL and female sex, fear of falling, higher stroke severity, diabetes mellitus or ischemic heart disease.

The mean EQ-VAS in our study was similar to the results from one prior study [21] of 328 stroke survivors with cerebral infarction at least 6 months after stroke onset, with a reported mean EQ-VAS of 66 (SD 21). However, compared to a sample of 2,870 individuals from the general population in Stockholm [22] with a mean age lower than 50 years and a mean rating scale score (the equivalent of EQ-VAS on a scale from 0 to 1.0) of 0.85, it was lower.

Our mean EQ-5D-3L index was higher than the 0.73 (SD 0.27) reported in a prior study [7] of 207 British stroke survivors at 60 months after a minor stroke (defined as a NIHSS score of 0–3). The British study consisted of younger subjects (mean age of 67.7 years) and had a lower proportion of women (37%). In fact, the mean EQ-5D-3L index in our study was just below what was found in the Swedish general population [22]. In a public health survey [22] of 3,069 citizens (20–88 years old) in Stockholm county, where 45.6% rated “perfect health” (i.e. had a health profile of 11111), the mean EQ-5D-3L index was 0.84.

Based on prior studies [7], HRQoL is indeed expected to be lower in stroke survivors than comparable controls at 5 years after stroke. However, HRQoL in stroke survivors has been reported to vary across different nations [23], independent of stroke severity, and such variations might partly explain the difference found.

The high proportion of stroke survivors who reported problems related to Pain/Discomfort using the EQ-5D-3L, which in our study was the most prevalent condition reported, follows the same pattern as in a Swedish public health survey [22]; Pain/Discomfort-related problems appear to be common among elderly people, regardless of whether they had a previous stroke or not. Furthermore, mild stroke survivors are known to experience long-term fatigue and anxiety [24] with potentially debilitating effects on long-term mobility, possibly contributing to our finding of reported problems related to Mobility, despite generally low NIHSS scores at baseline.

The association found between HRQoL and age and LOS is consistent with prior research [4, 5]. Longer in-patient stay is often related to more severe disease and might reflect the occurrence of complications like pneumonia or other infections. In a general Swedish population [22], women report worse health status than men. However, previous research has shown inconsistent evidence that female sex is a predictor of worse HRQoL in stroke survivors [25]. Proposed reasons for the occurrence of such a link have been that females are suffering stroke at older ages and that females are suffering worse physical impairment following a stroke [26]. Data from the current study suggest that female sex is not associated with impaired HRQoL several years after a stroke. On the contrary, there was a tendency of women scoring higher than men according to both EQ-5D-3L index and EQ-VAS.

The optimal goal of rehabilitation could be assumed to be a good HRQoL. A clinical implication based on the findings from the current study is that long-term follow-ups of individuals at risk of impaired HRQoL are important in order to, if possible, optimize long-term HRQoL.

The strengths of the current study include the consecutive inclusion at index stroke and the long follow-up time. However, our results should be understood within the context of their limitations. First, the large number of non-respondents reduced the population size and hence the power of the statistical analyses. It was expected that a proportion had died several years after a stroke. However, the fact that 58% of those who were eligible did not respond might have caused a selection bias, which could have influenced the validity of our findings. It is unclear why 20 participants did not answer the EQ-VAS scale completely. The high level of non-participants and missing data raises some concerns about bias. The non-participants were older, had more severe strokes and comorbidities which could have influenced their decision to participate. Secondly, since the patients requiring thrombolysis and thrombectomy were not included in this study, our findings are unlikely to be generalized to those patients who have received these treatments. Thirdly, the median NIHSS score at index stroke for the current population was on average low, indicating mild strokes. It was slightly lower than in the original cohort of 504 patients, who had a median NIHSS of 2 [8] and compared to Swedish national data [27], which reported a median NIHSS of 3. Furthermore, there was a relatively high proportion of posterior circulation syndrome (POCS) (35.8%), as classified using the Oxford Classification of Stroke, in comparison with prior research [28]. The existence of an association between HRQoL and subtype of stroke is possible; one prior study showed an association between reduced quality of life and a total anterior circulation stroke [29]. Based on those findings [29], despite using a different measure of quality of life, and the proportion of POCS in the current study, bias may exist. If such bias exists, it may account for underreporting of impaired HRQoL. There may also have been a selection at baseline that excluded the most severe cases of stroke from the original study due to lack of informed consent, and a selection of people with more mild strokes who participated in the follow-up. Therefore, the findings of this study cannot be generalized or valid to people other than people with mild strokes and living in the Western world. In addition, given the low proportion of hemorrhagic strokes, the results may not be generalizable to hemorrhagic strokes. Other independent variables could have been used as potential predictors. None of the selected variables associated with the dimension Anxiety/Depression.

This study provides insight into HRQoL and its risk factors, which could have implications for the planning of stroke rehabilitation. Future studies are suggested to direct their attention on possible benefits of implementing identified risk factors of impaired HRQoL in individualized rehabilitation plans.

## Conclusion

At 5 years after stroke, four out of five participants (79.1%) reported impaired HRQoL, mostly related to Pain/Discomfort. Higher age and longer hospital care period at index stroke were associated with impaired HRQoL related to the EQ-5D-3L dimensions Mobility, Self-Care and Usual Activities. The findings could assist to identify individuals at high risk of low HRQoL, who might benefit from special attention and psychological support.

## Data Availability

The data that support the findings of this study are available from the corresponding author upon reasonable request. According to Swedish regulations, permission to use data can be obtained after an application to and approval by the Swedish Ethical Review Authority.
